# The BERN Framework of Mind-Body Medicine: Integrating Self-Care, Health Promotion, Resilience, and Applied Neuroscience

**DOI:** 10.3389/fnint.2022.913573

**Published:** 2022-07-14

**Authors:** Tobias Esch, George B. Stefano

**Affiliations:** ^1^Institute for Integrative Health Care and Health Promotion, School of Medicine, Witten/Herdecke University, Witten, Germany; ^2^Center for Cognitive and Molecular Neuroscience, First Faculty of Medicine, Charles University, Prague, Czechia

**Keywords:** stress management, meditation, integrative medicine, behavioral medicine, salutogenesis, self-healing, placebo, mitochondria

## Abstract

**Background**: Mind-body medicine (MBM) focuses on improving our understanding of how the interactions between the brain, mind, body, and behavior can be used to promote health. In this narrative review, we present the basic principles of MBM, including the introduction of a rational framework for the implementation of MBM-based interventions. We also discuss the contributions of MBM to motivation and reward systems in the brain including those that may specifically involve the mitochondria.

**Results**: MBM can be used to promote health in patients with chronic diseases, especially conditions identified as lifestyle-related. MBM builds on salutogenesis, which is a paradigm that focuses on health (as opposed to disease) determinants and the development of individual resilience and coherence factors as a means to reduce stress, decrease the burden of disease, and improve the quality of life. This approach involves several well-known principles of self-healing and self-care. MBM interventions typically include behavioral modification techniques in conjunction with cognitive work focused on stress regulation, exercise, relaxation, meditation, and nutrition. We suggest the use of the acronym “BERN” (Behavior, Exercise, Relaxation, and Nutrition) to summarize the operational framework of this approach.

**Discussion**: Different BERN techniques act *via* shared autoregulatory central nervous system (CNS) reward and motivation circuitries. These systems rely on numerous neurobiological signaling pathways with overlapping effector molecules that converge, e.g., on nitric oxide (NO) as a common effector molecule. NO is critically coupled to reward physiology, stress reduction, and self-regulation as it modulates the responses of various mitochondrial, nuclear, and chromosomal processes within brain cells. NO has also been implicated in relevant outcomes (e.g., the placebo response).

**Conclusions**: MBM interventions typically follow the BERN model and aim to strengthen health and resilience, and reduce stress. The mechanisms of action of these processes involve the CNS reward systems and correlate with placebo and self-healing pathways.

## Background: Mind-Body Medicine

### Historical Perspective

Mind-body medicine (MBM) was founded by late Harvard cardiologist Herbert Benson in the context of modern meditation research that emerged in the 1970s (Benson and Klipper, 2000). Right from the start, there was the observation that connections and interactions between the brain, mind, body, and behavior can activate psychophysiological changes and a health-promoting potential in the individual—paths towards better health. As a generic term, mind-body medicine, from the start, included mental or behavioral medicine approaches and other techniques from the areas of exercise, relaxation, stress regulation, and nutrition (see below).

From the beginning, there was a close connection between the scientific investigation of meditation and relaxation mechanisms on the one hand and the exploration of individual self-healing and self-regulation potential on the other (Esch, 2014). After initial expeditions to the Himalayas and early research, e.g., on temperature regulation under meditation conditions in Buddhist monks, Herbert Benson founded the Mind-Body Medical Institute at Harvard Medical School in Boston in 1988 (today: Benson-Henry Institute for Mind Body Medicine). What Benson noticed early on was the observation that self-healing was a “mind thing” (Benson, 1987).

Meanwhile, mind-body and meditation phenomena such as self-induced blood pressure reductions or changes in peripheral body temperature, as well as changes in skin resistance or heart rate or heart rate variability in the context of relaxation, are known in many areas, including in biofeedback or autogenic training, as well as in self-hypnosis. Benson adopted this recurring physiological pattern in his concept of the relaxation response (Benson and Klipper, 2000)—the physiological antagonist of the biological stress response (Stefano et al., 2005), elicited and controlled by the central nervous system (CNS), i.e., its self-regulation and stress-relaxation axes.

The insights into the basic mechanisms and potential of self-regulation were not new at the beginning of mind-body medicine either, as this was based, among other things, on the research of the physiologist Walter B. Cannon, who decades earlier in the same laboratory (Cannon, 1933), in which Benson also worked, had done research on stress and regulation. What was new, indeed, was that people should be able to use mental techniques to influence the “involuntary” (i.e., autonomic) regulation in a targeted (i.e., conscious, focused) way. Therefore, Benson set out to make the study of such mind-body phenomena—and their possible significance for medicine—his life’s work under the new label of mind-body medicine (Komaroff, 2001). However, there was still a long way to go from the first investigations in the Himalayas to in-depth studies according to Western standards—including experimental human biological studies under laboratory conditions (e.g., Dusek et al., 2008).

### The Current State

Today, MBM has been widely implemented as an important component of general health care and medical practice in the United States, typically within the framework of behavioral medicine practices. MBM includes numerous straightforward and effective approaches that can be used to promote patient-centered health care (Esch, 2020). These approaches are conceptually and practically compatible with many current trends and disciplines in both clinical medicine and research (Dobos and Paul, 2019; Esch, 2020). Of note, MBM expands the outlook of somatically-oriented general medicine practices because it encourages healthcare providers to focus on behavioral and lifestyle orientation as a means to promote health (i.e., salutogenesis—see below) *via* self-help and self-healing skills (Esch, 2014, 2020).

National Institutes of Health (NIH), U.S. Department of Health and Human Services (2019) of the U.S. Department of Health and Human Services describe MBM as a discipline that focuses on:

• The nature of the interactions that link the brain, the body, the mind, and behavior to one another.
• How emotional, mental, social, spiritual, experiential, and behavioral factors can directly influence health.

National Center for Complementary and Integrative Health (NCCIH), National Institutes of Health (NIH), U.S. Department of Health and Human Services (2019) defines MBM techniques as those that:

• Focus on the mind as a means to influence physical functioning and promote health.
• Enhance individual capacity for self-knowledge and self-care.

MBM is thus based on the recognition of a central mind-body axis, which is a concept that encompasses consciousness, behavior, and the interactions between the brain and the body. A central principle of MBM focuses on how psychological, emotional, spiritual, social, experiential, and/or behavioral factors influence human health. Effective MBM techniques are those that support individual self-regulation of the mind-body connection based on these observations.

In this narrative review, we discuss underlying factors and overarching mechanisms associated with MBM, including a general consideration of motivation and reward systems in the CNS. We also present a rational framework for the various therapeutically-effective interventions that are typically used in MBM practice.

## Health Promotion and Salutogenesis

MBM is currently used clinically for primary prevention and health promotion as well as for the treatment of lifestyle-associated chronic diseases (Dobos et al., 2006). MBM can be used to address many of the problems that are most frequently presented to general practitioners (Laux et al., 2010), including, musculoskeletal complaints (e.g., pain disorders and chronic inflammatory/rheumatic diseases), lipid metabolism disorders, endocrine, metabolic, or nutrition-related diseases (including type 2 diabetes mellitus), high blood pressure, depressive symptoms, and/or gastrointestinal dysfunction (reviewed in Esch, 2020). MBM can also be used to support cancer treatment (Jeitler et al., 2017; Voiss et al., 2019), as an adjunct therapy for addiction treatment, strengthening resilience, and stress reduction (Esch, 2008).

In contrast to psychosomatic medicine (Fava and Sonino, 2010), MBM is not primarily linked to psychopathology or the presence of a specific disorder. While MBM techniques can be used to address a specific indication, they can also be applied more generally as a means to promote health and well-being. Similarly, and unlike conventional psychotherapy, the primary goal of MBM is not the uncovering and clarification of psychological conflicts; no psychodynamic explanations are typically sought for behaviors that need to be addressed (Paul and Altner, 2019). MBM interventions are primarily aimed at developing health-promoting attitudes and healthy, sustainable behaviors in everyday life. This approach is based on the concept of salutogenesis, which is the assumption that, in addition to factors that trigger or sustain disease processes, one can also focus on factors that generate and/or maintain human health. These “health protection factors” or “resistance resources” include those focused on stress reduction (Antonovsky, 1979, 1996).

## The Bern Framework

MBM is based on the principle that all individuals have the potential for self-healing and can be trained to accomplish this within the framework of a resource-oriented and integrative (i.e., salutogenic) medicine program (Esch, 2002; Dobos et al., 2006; McClafferty, 2011; Brinkhaus and Esch, 2021). MBM interventions focus on the individual and consider individual competencies. The terms “auto-” or “self-regulation” or “self-regulatory medicine” are often used to describe the underlying mechanisms and active factors involved in this process (Esch, 2003, 2014, 2017, 2020).

**Figure 1:**
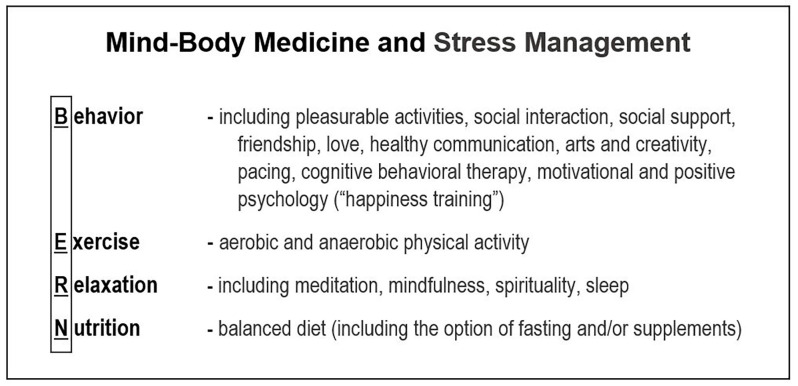
The BERN framework of mind-body medicine (MBM). The four principles of integrative multimodal MBM and stress management programs include Behavior, Exercise, Relaxation, and Nutrition.

MBM-based interventions have been established that follow the “BERN” framework (Figure 1). As a group, these are strategies that have an impact on behavior (B), exercise (E), relaxation (R), and (N) nutrition (Esch, 2008). Within this context, it is important to note that BERN is not a distinct program or a single example of a set of mind-body interventions (MBIs), but rather a description of a framework that encompasses numerous multimodal interventions. BERN represents a practical set of tools that are based on the individual therapeutic pillars that define MBM and MBIs.

Other important components of the multifactorial BERN approach include social support, which is usually included in the behavior column, and spirituality, faith/belief, meditation, or mindfulness techniques, which are usually associated with relaxation (Esch, 2008; Jeitler et al., 2017). Additional behavioral aspects enclose resilience factors (Ludolph et al., 2019) and instruments that promote “positive psychology” (Seligman and Csikszentmihalyi, 2000; Siegel, 2009; Esch, 2017). Likewise, other elements used to promote relaxation include formal meditation and mindfulness exercises.

Although MBM is still relatively young and the framework presented here was developed in a modern medical-scientific context, it must not be forgotten that individual components (columns, pillars, specific techniques) have a very long tradition in medicine. Hence, we can already find essential parts of them in Hippocrates and Galen (van der Eijk, 2011; Esch, 2014). The combination—practically the whole package of complete BERN-MBIs—has been around for a long time: in yoga, for example, the multifactorial practice of posture, movement, relaxation (meditation, breathing exercises), nutrition and even cognitive-mental techniques (visualizations, affirmations, etc.) was implemented long ago, and this “BERN” framework is also showing impressive effectiveness in modern medicine (Cramer et al., 2017; Shin, 2021; Wibowo et al., 2022).

## Bern and The “Two-Door Model” of Behavioral and Mind-Body Medicine

The BERN framework of MBM promotes the general principles of resilience and health maintenance (see also below). The behavior-related health and lifestyle modification programs are typically introduced as components of a preventive, behavioral, or complementary medical intervention. However, the actual implementation of BERN is frequently handled not by a physician, but by health-wellness coaches, MBM instructors, behavior therapists (frequently psychologists), or specifically-trained individuals with expertise in health promotion strategies (Werdecker and Esch, 2021). In other words, these strategies are typically implemented *via* a “two-door” model (Werdecker and Esch, 2021; Esch, 2021a). Patients are initially seen in an outpatient facility where a physician provides advice and treatment for a disease process, and an allied therapist or MBM trainer then discusses—and performs—health promotion and potential behavioral changes designed to target individual health and self-healing potentials (Werdecker and Esch, 2021).

This dichotomy is not just for pragmatic and capacitive reasons, e.g., to preserve resources. Irrespective of the need for the MBM instructors to demonstrate their own professionalism, training, and qualifications for the effective implementation of BERN/MBM coaching, there is also a paradigmatic reason for the “two-door” model: doctors are well-trained experts for detecting and treating pathologies, risks, and diseases. Indeed, the external doctor (the “medicus”) should be able to carry out and implement the best possible diagnostics and therapy as well as preventive measures. However, this pathogenetic view differs fundamentally—i.e., paradigmatically—from the perspective of the inner doctor (the “archaeus”), which addresses the inner potential for self-healing and salutogenesis: BERN and MBM as frameworks and set of techniques or practical programs for health promotion and empowerment of the inner doctor are intended, as outlined, to increase individual health potentials, i.e., to generally activate health protection factors, increase resistance resources, and reduce allostatic loads, i.e., stress (McEwen, 1998; Esch, 2002, 2014). This salutogenetic perspective is complementary—not alternative or exclusive—to the pathogenetic approach as it prevails in conventional medicine (Esch and Brinkhaus, 2021). The combination of both perspectives—in theory, and practice—may also be called “integrative medicine” (Brinkhaus and Esch, 2021).

## Bern and Resilience

There has been a sizable increase in the amount of evidence available that supports the use of MBM in health promotion as well as in the prevention and therapy of various diseases, especially in primary care settings (Esch and Brinkhaus, 2021). There are now numerous publications and systematic reviews that support the efficacy of MBM-based interventions (e.g., Astin et al., 2003; Anderson and Taylor, 2011; Prochaska and Prochaska, 2011; Cramer et al., 2015). However, while robust evidence is available to support the four BERN pillars (see below), several issues remain unclear. For example, additional evidence will be needed for an effective evaluation of MBM and its role in preventing or promoting more favorable outcomes in diseases with high mortality.

Resiliency (or: resilience) is the ability to maintain adaptive functioning in response to the ongoing stress of daily living (Park et al., 2021). Hence, resiliency training is a core element of MBM (Stahl et al., 2015; Esch and Esch, 2021; Park et al., 2021), and here, it typically refers to practicing a set of core coping skills (i.e., relaxation, stress awareness and management, and adaptive/behavioral strategies). However, evaluating the overall health outcomes of MBM-resilience practice in specific diseases and patient populations remains a challenge, e.g., due to a significant lack of longitudinal and controlled studies.

Among the issues associated with these assessments, the classifications (i.e., the assignment of a given attribute to a specific BERN pillar) are frequently not uniform. For example, some researchers assign the resilience factors solely to the areas associated with cognition and behavior (B) vs. relaxation (R) and mindfulness (Stahl et al., 2015). However, some combine B and R into their own synoptic resilience framework (Ludolph et al., 2019). Others see physical training and sport as a formal reference for resilience training, and thus assigned to Exercise (E), or the entire BERN framework (Komaroff, 2001; Stahl et al., 2015).

Moreover, it is not yet clear whether individual BERN or lifestyle factors can be fundamentally influenced by therapy; this remains a subject of intensive research with sometimes controversial results. At this time, there is strong evidence supporting the positive impact of exercise (E) or physical activity (e.g., Miko et al., 2020; Posadzki et al., 2020) and nutrition-related (N) interventions (e.g., Rees et al., 2013; Naude et al., 2017), such as in cardiovascular health (e.g., Casas et al., 2018; Fiuza-Luces et al., 2018), sometimes also used in combination (e.g., Nitschke et al., 2022). Likewise, the evidence supporting a role for primarily cognitive or psychological techniques (B) has improved significantly in recent years, despite methodological weaknesses and the substantial heterogeneity associated with this field of study (reviewed in Esch, 2002, 2017, 2020). Recent meta-analyses that focus on positive psychology, resilience, and optimism have generated an overall positive picture (Bolier et al., 2013; Chakhssi et al., 2018; Joyce et al., 2018; Rozanski et al., 2019). These findings also include studies on risk stratification in which the results associated with different pillars are combined (e.g., Chomistek et al., 2015). For the relaxation (R) or meditation pillar, too, the evidence appears rather robust, although weaker overall (e.g., Astin et al., 2003; Ospina et al., 2007; Goyal et al., 2014; Stahl et al., 2015; Long et al., 2017; Flynn, 2018; Michaelsen et al., 2021). Current knowledge that addresses molecular mechanisms and active factors (e.g., Esch and Stefano, 2004; 2010; Esch et al., 2004; Stefano and Esch, 2005; Salamon et al., 2006; Stefano et al., 2006, 2019a; Esch, 2013; Esch et al., 2018, Esch et al., 2020a; also see below), as well as its inherent cost-efficiency (Sobel, 2000), has also promoted a more widespread acceptance of MBM in the healthcare systems in many countries.

## The Neurobiological Basis of Mind-Body Medicine

Current research on the neurobiological basis of MBM, including the molecular and autoregulatory pathways underlying the positive responses to relaxation and meditation, has shown relevant parallels to physiologic activation patterns that are similar to those associated with, for example, placebo mechanisms (Moss et al., 2022; Sezer et al., 2022). This applies in particular to the involvement of neurobiological reward processes, including the specific reward and motivation systems that are initiated in the three limbic levels of the CNS (Esch and Stefano, 2004, 2010; Esch et al., 2004, 2017; Esch, 2013, 2017; Esch, 2020).

Interestingly, the existence of a placebo effect was disputed for a long time or, alternatively, dismissed as a methodological error (Esch, 2014, 2020). Today, the placebo effect is widely recognized and accepted, although it is not yet fully understood. The results from studies published by Kam-Hansen et al. (2014) and Kaptchuk et al. (2010) suggested that the placebo effect exists even in studies involving “open-label treatments” (i.e., when the patient is informed that he or she will be taking a drug with no active ingredients). This study design virtually eliminates concerns regarding deception. However, it is critical to note that these actions may have a positive influence on self-healing, as the possibility of self-regulation remains functional under these circumstances. Perhaps equally interesting, as predicted earlier by anthropologists (as described in Esch, 2014, 2020), recent research has uncovered genetic factors that contribute to the susceptibility to the placebo effect (e.g., Hall et al., 2012).

**Figure 2:**
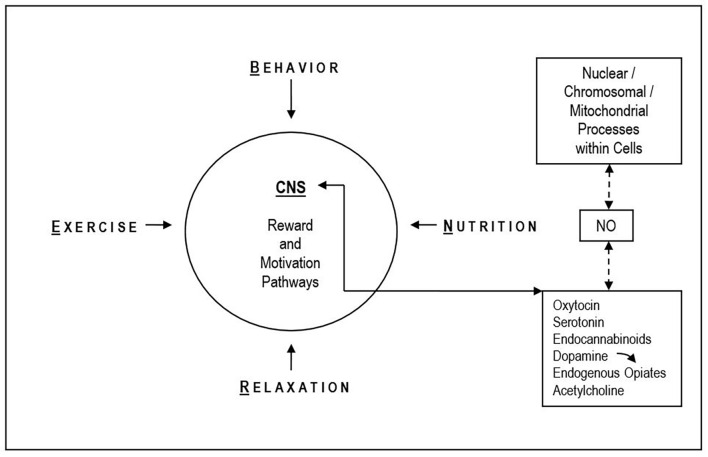
The shared neurobiology of BERN approaches. Different BERN techniques and MBM (self-care) practices may have a direct impact on physiologic regulatory reward and motivation circuitries in the central nervous system (CNS). This commonality may represent overlapping functions associated with the general neurobiological principles of autoregulation (i.e., the potential for self-healing). MBM engages numerous CNS signaling pathways and effector molecules to promote healing, many of which converge on nitric oxide (NO) as an example for a common effector (i.e., a secondary or tertiary messenger). NO is critically coupled to the reward physiology and stress reduction/self-regulation and has a decisive impact on various processes within the cells and their organelles (i.e., chromosomes, nucleus, mitochondria). NO has also been specifically implicated in pathways leading to the placebo response (for further information and references: see text).

Accordingly, the placebo effect also works if it is not based on deception or “blind belief”. The intentional directing of attention to a previously learned (conditioned)—and positively anticipated (expected), in a suitable situation—favorable outcome activates self-healing processes (Esch, 2014). It is undisputed that there can also be specific effects within the framework of MBM and MBIs, just as the individual pillars of the BERN framework also present specific components in addition to overlapping signaling paths (see Esch and Stefano, 2010; Figure 2). In addition to the anticipation and motivation systems involved, other CNS networks also come into play, such as the salience network, which is closely linked to the reward system, the resting state network (default mode network), or parts for self-processing and self-reference, and finally, frontal CNS networks for executive functions (Lee et al., 2018; Gothe et al., 2019; Esch, 2021b; Michaelsen and Esch, 2021, 2022; Zhang et al., 2021). The feeling of non-wanting or of relief (i.e., stress reduction), as for “happiness”, is also embedded here (Esch, 2022). However, placebo effects may account for and serve as blueprints (or “relay stations”) for the most relevant CNS networks and functions involved in MBIs and BERN practices and their reported health outcomes.

Hence, as suggested, also by the results of modern analysis and imaging methods, the brain is the most likely source of all self-regulatory responses. The activation of these phenomena is accompanied by the release of characteristic CNS messengers (e.g., dopamine). The regions and networks activated are located in phylogenetically old areas of the CNS, including the limbic reward regions (Figure 2; Esch et al., 2004; Esch and Stefano, 2004, 2010; Michaelsen and Esch, 2021). Many self-healing techniques have been associated with these same processes; while the factors that activate each mechanism may be specific to each individual based on strong genetic and/or cultural influences, the overall mechanism may rely on more universal biological principles (Esch, 2014, 2020).

Thus, it is not surprising to find overlap or convergence of many different self-healing properties on a central autoregulatory center, ultimately leading to a reduction of inflammation and stress (Esch and Stefano, 2004, 2010; Stefano et al., 2005). Both, BERN and the placebo effect, seem to engage reward and motivation centers in the CNS (Figure 2; Stefano et al., 2001; Esch and Stefano, 2004; Esch et al., 2004; Fricchione and Stefano, 2005; Esch, 2014, 2020).

Overall, we can conclude that both MBM and the placebo effect are based on a system of self-regulation, and require the activation and functioning of a corresponding biological CNS reward-motivation system. Effective activation of this system relies on the convergence of factors associated with an “embedded” (individually memorized or culturally learned) positive experience. This experience leads to positive expectations for the future (i.e., anticipation) based on a “suitable opportunity” that depends directly on concrete conditioning and also on the specific context. *Via* this mechanism, a positive outcome is anticipated and the regulatory processes are focused in this direction. Correspondingly, the newly-focused attention frame does not anticipate other outcomes. In other words, one “trusts” that there will be a repetition of the positive result that has already been experienced in the past.

In principle, these outcomes can be achieved *via* the actions of brain regions or networks that represent a systemic or functional unit (Esch and Stefano, 2004; Esch, 2017). With respect to the person affected (i.e., the “regulator”), this implies that healing is possible and lies within the intentional window of perception and probability. While specific methodologies, for example, the BERN framework, provide context for these phenomena, these are frequently determined by cultural and/or situational attributes. In these cases, positive conditioning can provide self-assurance and may lead to an expectation of positive self-efficacy. Once complete, the result is that one can experience oneself as competent and effective (Esch, 2003).

In a sense, we can understand MBM and related behavioral self-healing rituals as practical anchors of the neurobiological and psychological connections presented (Esch and Stefano, 2010). Thus, MBM can be viewed as an “applied placebo effect”, and self-healing as a type of “placebo medicine” (Stefano et al., 2001). As described above, results from several recent neurobiological studies stand in strong support of the involvement of relevant brain regions for limbic and reward regulation in self-healing and MBM practices (Moss et al., 2022; Sezer et al., 2022).

In summary, the concepts and phenomena associated with self-regulation, BERN, and MBM are of great relevance to medicine (Esch, 2014). The benefits of these phenomena will be revealed by additional research, self-understanding, and clinical application.

## Hypothesis for Future Research: Additional Pathways and Mitochondrial Involvement

Stress management and self-regulation training usually consist of one to all of the following instruments and activities: behavioral or cognitive, exercise, relaxation, and nutritional or food interventions (BERN), including social support and spirituality. Some of the relevant molecular regulatory pathways involved are already known, while others remain to be described.

Based on the observed physiological and molecular mechanisms underlying the known responses to MBM, we further hypothesize that interventions involving relaxation and other modalities act by modulating autonomic responses (Stefano et al., 2019a, b). This hypothesis also suggests that MBM behavioral training programs such as BERN have an overall positive impact on mitochondrial bioenergetics and insulin secretion and can reduce the activation of pro-inflammatory and stress-related pathways. We surmise that a plausible model of behaviorally-mediated regulation of whole-body metabolic processes must be intrinsically broad-based and multifaceted, and this will require the integration of numerous contributions from functionally interactive organs associated with both the peripheral and CNS (Stefano et al., 2019a, b).

Beneficial behaviors and strategies to overcome stress are, as a more general principle, neurobiologically rewarded by pleasure (“happiness”) induction, yet positively and physiologically amplified and reinforced (Esch et al., 2003; Michaelsen and Esch, 2021; Esch, 2022). The underlying physiology (i.e., self-/autoregulation) seems to work *via* dopamine, endocannabinoid, endorphin, and morphine release, as well as stress hormone (adrenaline/noradrenaline, cortisol) modulation, oxytocin and serotonin signaling pathways (Esch et al., 2002, 2004; Esch and Stefano, 2004, 2010; Esch, 2022), many of which act *via* nitric oxide (NO) release—apart from other messenger molecules (Figure 2). These latter effects are unspecific, however, downregulatory and clearly stress-reducing by their nature, acting *via* local CNS networks.

States of persistent activity within local circuits of cortical neurons are tonically modified by inhibitory synaptic depression. Importantly, gamma-aminobutyric acid (GABA) CNS systems, exemplified in this regard, are well established to represent the major source of regulatory neural inhibition on chronically active local circuit cortical neurons (Mann et al., 2009). The inhibitory/suppressive effects of GABA are pharmacologically mediated by differential activation of GABAA and GABAB receptor subtypes (Mann et al., 2009). Hence, the clinical importance of restorative GABAergic transmission in anesthesia, pain and analgesia, cardiovascular function, and psychiatry is supported by the wide variety of pharmaceutical agents designed to selectively target GABAA and GABAB receptor subtypes (e.g., Hepsomali et al., 2020). The essential regulatory effects of GABA on patterns of excitatory activity across cortical microcircuitry extend to GABAergic modulation of brainstem neurons involved in CNS mediation of autonomic control of cardiovascular function within the nucleus tractus solitaries (Tjen-A-Looi et al., 2014). In sum, the putative regulatory effects of GABAergic transmission on multiple physiological aspects of the relaxation response are compelling and most likely extend to integrated patterns of neuronal activities throughout sensory, cognitive, and autonomic regulatory neuronal groupings throughout the CNS (Stefano et al., 2019a; Hepsomali et al., 2020; Büttiker et al., 2021; Namgung et al., 2021).

In addition, we further speculate that the activation of numerous protective and anti-bio-senescence processes may have emerged during evolutionary development to ensure the survival of hybrid prokaryotic/eukaryotic progenitor cells (i.e., the precursors of mitochondria) and protect them from the harmful byproducts of oxidative metabolism (Stefano et al., 2019a, b; Esch et al., 2020b). Preservation and adaptation of multifaceted regulatory molecules, notably NO, most likely paralleled the development of eukaryotic cells *via* multifaceted stereo-selective recognition and conformational matching executed by complex biochemical and enzyme systems.

Hence, the relaxation response may be a manifestation of a synchronized process of molecular metabolic corrective responses that also include cognition (i.e., “awareness”; see Stefano et al., 2019a, b). In fact, cognitive-behavioral, as well as relaxation, meditation, and other BERN practices can show an intimate connection to intracellular processes and signaling, including mitochondrial and chromosomal activation (Figure 2; also see Esch et al., 2018, 2020b; Stefano et al., 2019a,b). In this regard, as stated earlier, complex BERN and other mind-body techniques such as yoga have already demonstrated their neurobiological, psychoneuroendocrinological, and -immunological as well as physiological stress-reducing and health-promoting potential (Michalsen et al., 2005; Wolever et al., 2012; Posadzki et al., 2014; Cramer et al., 2015, 2017; Pascoe et al., 2017; Shin, 2021; Koch et al., 2022; Wibowo et al., 2022).

## Conclusions

MBM is a field that focuses on health promotion, prevention, and treatment of lifestyle-related diseases. It builds on the concepts of salutogenesis and resilience, based on principles that include general self-healing or self-care. MBM interventions follow the BERN framework (behavior, exercise, relaxation, and nutrition). The responses to various BERN techniques converge on shared mechanisms of CNS autoregulation involving limbic reward and motivation systems. Various neurobiological signaling pathways and effector molecules within these systems overlap and converge on constitutive NO as a common effector molecule. Other key regulators—such as GABA—are modulated as well, resulting in an overall downregulating potential for MBM and BERN. Thus, NO—and related messenger substances—are critically coupled to the reward physiology as well as stress reduction and self-regulation, and they may have an impact on mitochondrial, nuclear, and/or chromosomal processes as well as the placebo response.

## Author Contributions

All authors equally contributed to planning/designing, writing, and reviewing the manuscript. All authors contributed to the article and approved the submitted version.

## Conflict of Interest

The authors declare that the research was conducted in the absence of any commercial or financial relationships that could be construed as a potential conflict of interest.

## Publisher’s Note

All claims expressed in this article are solely those of the authors and do not necessarily represent those of their affiliated organizations, or those of the publisher, the editors and the reviewers. Any product that may be evaluated in this article, or claim that may be made by its manufacturer, is not guaranteed or endorsed by the publisher.
